# Does murine spermatogenesis require WNT signalling? A lesson from *Gpr177* conditional knockout mouse models

**DOI:** 10.1038/cddis.2016.191

**Published:** 2016-06-30

**Authors:** Su-Ren Chen, J-X Tang, J-M Cheng, X-X Hao, Y-Q Wang, X-X Wang, Y-X Liu

**Affiliations:** 1 State Key Laboratory of Stem Cell and Reproductive Biology, Institute of Zoology, Chinese Academy of Sciences, Beijing, China; 2 University of the Chinese Academy of Sciences, Beijing, China

## Abstract

Wingless-related MMTV integration site (WNT) proteins and several other components of the WNT signalling pathway are expressed in the murine testes. However, mice mutant for WNT signalling effector *β*-catenin using different Cre drivers have phenotypes that are inconsistent with each other. The complexity and overlapping expression of WNT signalling cascades have prevented researchers from dissecting their function in spermatogenesis. Depletion of the *Gpr177* gene (the mouse orthologue of *Drosophila Wntless*), which is required for the secretion of various WNTs, makes it possible to genetically dissect the overall effect of WNTs in testis development. In this study, the *Gpr177* gene was conditionally depleted in germ cells (*Gpr177*^flox/flox^, *Mvh*-Cre; *Gpr177*^flox/flox^, *Stra8*-Cre) and Sertoli cells (*Gpr177*^flox/flox^, *Amh*-Cre). No obvious defects in fertility and spermatogenesis were observed in these three *Gpr177* conditional knockout (cKO) mice at 8 weeks. However, late-onset testicular atrophy and fertility decline in two germ cell-specific *Gpr177* deletion mice were noted at 8 months. In contrast, we did not observe any abnormalities of spermatogenesis and fertility, even in 8-month-old *Gpr177*^flox/flox^, *Amh*-Cre mice. Elevation of reactive oxygen species (ROS) was detected in *Gpr177* cKO germ cells and Sertoli cells and exhibited an age-dependent manner. However, significant increase in the activity of Caspase 3 was only observed in germ cells from 8-month-old germ cell-specific *Gpr177* knockout mice. In conclusion, GPR177 in Sertoli cells had no apparent influence on spermatogenesis, whereas loss of GPR177 in germ cells disrupted spermatogenesis in an age-dependent manner via elevating ROS levels and triggering germ cell apoptosis.

WNT signalling is a highly conserved cell-to-cell communication mechanism that consists of a canonical (WNT/*β*-catenin pathway) and noncanonical branch (reviewed in Logan and Nusse^1^). WNT signalling has essential functions in development and tissue homoeostasis, and the misregulation of WNT signalling has been implicated in several pathological states (reviewed in Cadigan^2^). The vertebrate WNT family consists of 19 secreted cysteine-rich glycoproteins, among which WNT1,^3^ WNT3,^4^ WNT3a,^5^ WNT4,^6^ WNT5a,^7^ WNT7a,^8^ WNT10b^9^ and WNT11^10^ have been reported in the developing testes or in the testes of adult male rodents and humans. Several other components of the canonical WNT signalling pathway, such as DVL1,^11^ FZ9,^12^ *β*-catenin,^13^ NKD1,^14^ DKKL1^15^ and APC,^16^ have also been detected in testis tissues.

Gene knockout mouse models have provided information about WNTs and WNT signalling. During the embryonic stage of mouse development, WNT3 and WNT3A regulate primordial germ cell (PGC) specification and early expansion.^17, 18^ WNT5A-ROR2 serves as an important cue for PGC migration; loss of *Wnt5a* disrupts PGC migration into the genital ridge.^19, 20^ It is also well established that WNT signalling mainly plays a negative role in testis determination and proper repression of WNT signalling by the SRY/SOX9/FGF9 pathway is important for normal sexual differentiation.^21, 22^ Constitutive activation of WNT signalling effector *β*-catenin in Sertoli cells causes male infertile phenotypes, including testis cord disruption, inhibition of Müllerian duct regression and germ cell apoptosis.^23, 24^ Further study suggests that constitutive activated *β*-catenin signalling in Sertoli cells downregulates spermatogonial stem cell (SSC) activity via the paracrine factor WNT4.^25^ In contrast, Sertoli cell-specific knockout of *β*-catenin causes no detectable abnormalities.^23^ WNT3A/*β*-catenin signalling was reported to stimulate proliferation, morphological changes and cell migration of a spermatogonial cell line *in vitro*.^5, 9^ WNT5A, secreted from Sertoli cells, has been shown to support SSC maintenance through *β*-catenin-independent JNK signalling.^7^ Post-meiotic male germ cell-specific deletion of *β*-catenin using *Prm1*-Cre results in significantly reduced sperm count, increased germ cell apoptosis and impaired fertility.^26^ However, Rivas *et al.* suggested that conditional deletion of *β*-catenin using *Stra8*-Cre (express only in males beginning at postnatal day 3^27^) has no effect on male fertility.^28^ In a recent article,^29^ Takase *et al.* reported that WNT6 secreted by Sertoli cells activates WNT/*β*-catenin signalling in undifferentiated spermatogonia, including SSCs, which mediate the proliferation but not the maintenance of undifferentiated spermatogonia.

Collectively, previous studies have suggested that WNT signalling and WNTs play multiple roles in spermatogenesis, but there are still the following problems: (1) the conclusions about the function of WNT/*β*-catenin in germ cells are inconsistent with each other;^26, 28, 29^ (2) the overlapping expression pattern of the various WNTs and their functional redundancy obscure the true consequences of removing individual *Wnt* genes; and (3) *β*-catenin does play a central role in the canonical WNT pathway, but it also serves as a membrane protein of the cell junction complex.^30^

WNTs are secreted as glycoproteins from WNT-producing cells into the extracellular milieu.^31^ In 2006, Bänziger *et al.*^32^ and Bartscherer *et al.*^33^ identified a novel WNT pathway component, Wntless (WLS), and showed that it is responsible for the secretion of WNT proteins from signalling cells. Loss of WLS function has no effect on other signalling pathways, but it appears to impede all of the WNT signals.^32, 33^ Retromer retrieves endocytosed WLS from endosomes and recycles it back to the trans-Golgi network for its further function in WNT secretion.^34^ Accordingly, the conditional knockout (cKO) mouse models of *Gpr177*, the mouse orthologue of *Drosophila Wls*, is more appropriate than other established models related to *β*-catenin for the study of the role of WNT singalling (both canonical and noncanonical) and WNTs.

Mice homozygous for *Gpr177* (*Gpr177*^−/−^) die in the embryonic stage due to defects in body axis establishment.^35^ Carpenter *et al.*^36^ generated mice with a conditional null allele for *Gpr177* (recombination of the loxP sites using Cre resulting in the removal of exon 1) and showed that GPR177 is essential for the development of brain and pancreas. Fu *et al.*^37^ created a novel conditional *Gpr177* knockout mouse line (loxP sites flanking exon 3) and observed that the loss of GPR177 in WNT1-expressing cells causes mid/hindbrain and craniofacial defects, which resemble the double knockout of WNT1 and WNT3a as well as *β*-catenin deletion in the WNT1-expressing cells. Zhu *et al.*^38^ generated a different *Gpr177* cKO mouse line carrying an exon 3-floxed allele and showed that GPR177-mediated WNTs regulate early patterning along the three axes of the limb bud and also sustain cell proliferation and survival of distal limb mesenchyme. Sebsequently, these *Gpr177* cKO mouse lines have been utilised to investigate the roles of WNT signalling and WNTs in a variety of tissues, such as embryonic hair follicles, fungiform placodes and teeth.^39, 40, 41, 42^

Because the *Gpr177* mRNA level is expressed in mouse testis^43^ and the role of WNT signalling in spermatogenesis is still unclear, we generated and analysed germ cell-specific (*Gpr177*^flox/flox^, *Mvh*-Cre and *Gpr177*^flox/flox^, *Stra8*-Cre) and the Sertoli cell-specific (*Gpr177*^flox/flox^, *Amh*-Cre) *Gpr177* cKO mice. We observed that selective loss of *Gpr177* in germ cells or Sertoli cells blocks the secretion of cell-specific WNT ligands. GPR177 in Sertoli cells has no apparent influence on spermatogenesis, whereas germ cell-specific *Gpr177* deletion mice exhibit an age-dependent reproductive phenotype: fertile when young and subfertile when older. We further suggest that oxidative stress is involved in age-dependent spermatogenic damage of germ cell-specific *Gpr177* deletion mice.

## Results

### GPR177 expression in mouse testes

**Figure 1:**
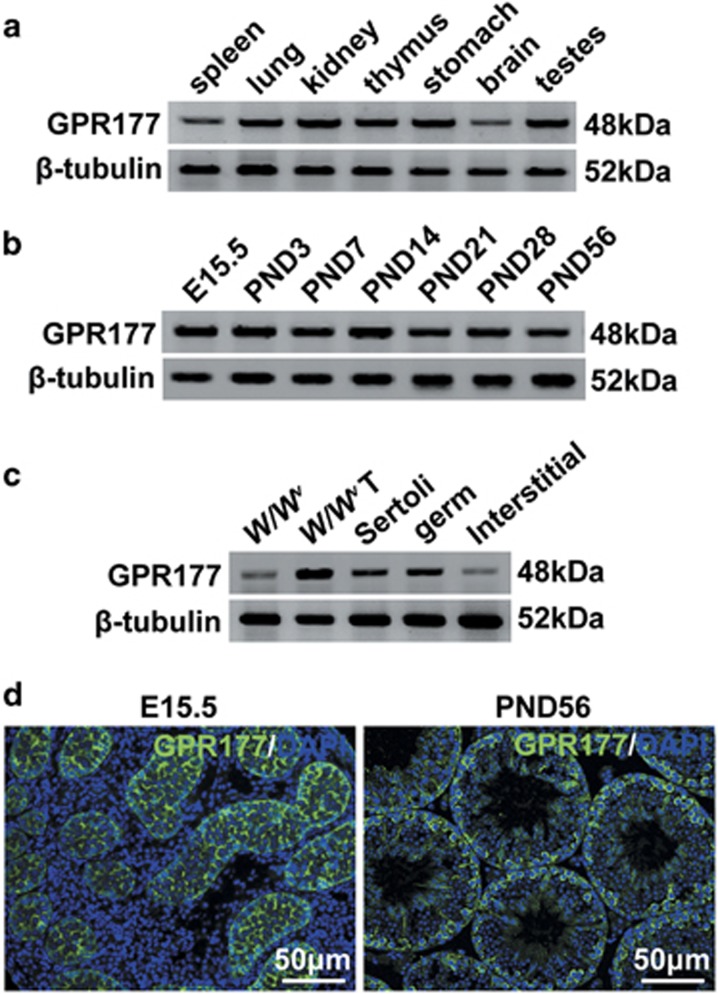
GPR177 protein level and localisation in mouse testes. (**a**) Expression of GPR177 in mouse tissues, including spleen, lung, kidney, thymus, stomach, brain and testes by western blot. (**b**) Expression of GPR177 in testes at different developmental stages, including E15.5, PND 3, 7, 14, 21, 28 and 56. (**c**) Protein level of GPR177 in *W/W*^*v*^ testes, *W/W*^*v*^ recipient testes after SSC transplantation, and isolated Sertoli, germ and interstitial cells. *β*-Tubulin served as a protein loading control in (**a**–**c**). (**d**) The cellular localisation of GPR177 in sections of testes from E15.5 and PND56 mice was displayed by immunofluorescent staining. The nuclei are counterstained with DAPI (blue). The images shown were representative results of experiments that were repeated three times using samples from different sets of testes, which yielded similar results. Scale bars, 50 *μ*m

The findings of a previous study suggest that *Gpr177* mRNA is expressed ubiquitously.^43^ In this study, we observed that GPR177 was expressed in many mouse tissues, including the spleen, lung, kidney, thymus, stomach, brain and testes, using western blot analysis (Figure 1a). Furthermore, the protein level of GPR177 in testis did not obviously differ between embryonic day (E) 15.5 and postnatal days (PND) 3, 7, 14, 21, 28 and 56 (Figure 1b). To evaluate GPR177 expression in different testicular cells, we assessed the GPR177 protein level in *W/W*^*v*^ testes (lacking endogenous germ cells), *W/W*^*v*^ recipient testes after SSC transplantation, freshly isolated Sertoli cells, germ cells and interstitial cells from adult mouse testes. GPR177 was highly expressed in germ cells and Sertoli cells, with little expression in interstitial cells (Figure 1c). The expression level of GPR177 protein was higher in *W/W*^*v*^ recipient testes after SSC transplantation than in *W/W*^*v*^ testes (Figure 1c). Immunofluorescence staining in E15.5 and PND56 testes further showed that GPR177 was visibly present in several testicular cell types, including germ cells and Sertoli cells (Figure 1d).

### Efficient and specific disruption of *Gpr177*

**Figure 2:**
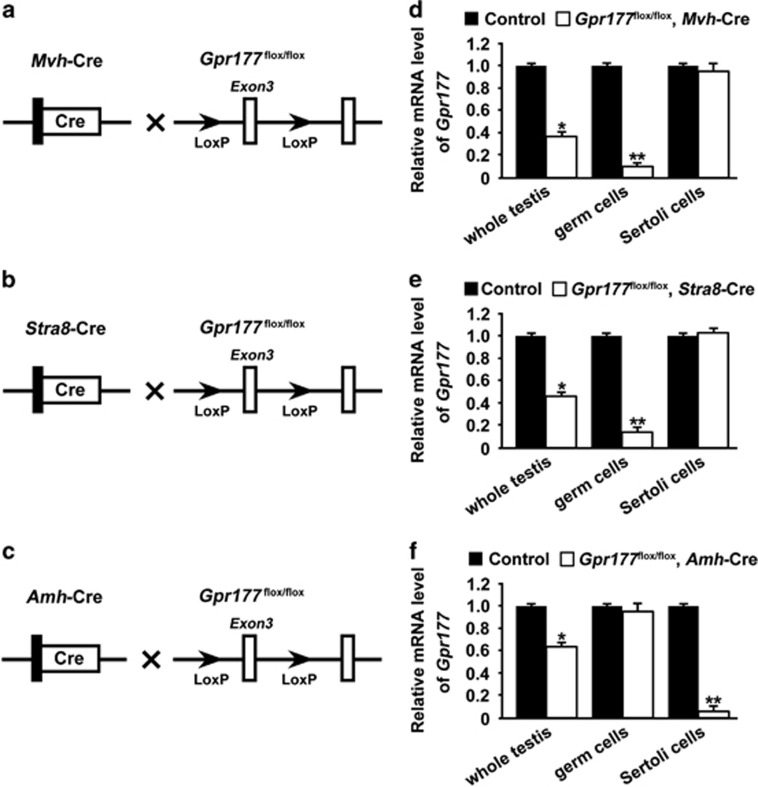
Targeted disruption of the *Gpr177* gene. (**a**–**c**) Hybrid scheme used to develop *Gpr177* cKO mice. Mice carrying a targeted *Gpr177* allele (LoxP sites flank exon 3 of the *Gpr177* allele) were crossed with *Mvh*-Cre or *Stra8*-Cre or *Amh*-Cre transgenic mice to selectively delete *Gpr177*. The gene knockout was confirmed by PCR genotyping. The genomic DNA isolated from the mouse tails was amplified with primer pairs specific for the wild-type (+) (~100 bp) and flox alleles (~200 bp) or different Cre bands (*Mvh*-Cre: 240 bp; *Stra8*-Cre: 326 bp and *Amh*-Cre: ~100 bp). (**d**–**f**) qRT-PCR analysis showing the conditional loss of *Gpr177* mRNA in total testis extracts, germ cell or Sertoli cell extracts of three *Gpr177* cKO mice. *Gapdh* served as the internal control gene. The data are expressed as the mean±S.E.M. *P<0.05, **P<0.01

To assess the cell type-specific function of GPR177 during spermatogenesis, we generated mice in which the *Gpr177* gene was specifically disrupted in germ cells using *Mvh*-Cre (Figure 2a) or *Stra8*-Cre (Figure 2b) and Sertoli cells using *Amh*-Cre (Figure 2c). Genotyping of the mice was performed by PCR using specific primers to distinguish wild-type or floxP alleles and different Cre bands. *Gpr177* deletion efficiency in germ cells and Sertoli cells was assessed by detecting the *Gpr177* mRNA level in testis, germ cells and Sertoli cells. As expected, we observed a significant reduction in *Gpr177* mRNA levels in both whole testis lysate and isolated germ cells from *Gpr177*^flox/flox^, *Mvh*-Cre (Figure 2d) and *Gpr177*^flox/flox^, *Stra8*-Cre testes (Figure 2e). Similarly, the *Gpr177* mRNA level was significantly reduced in both whole testis lysate and isolated Sertoli cells from *Gpr177*^flox/flox^, *Amh*-Cre testes (Figure 2f).

### GPR177 is responsible for secretion of WNT proteins

**Figure 3:**
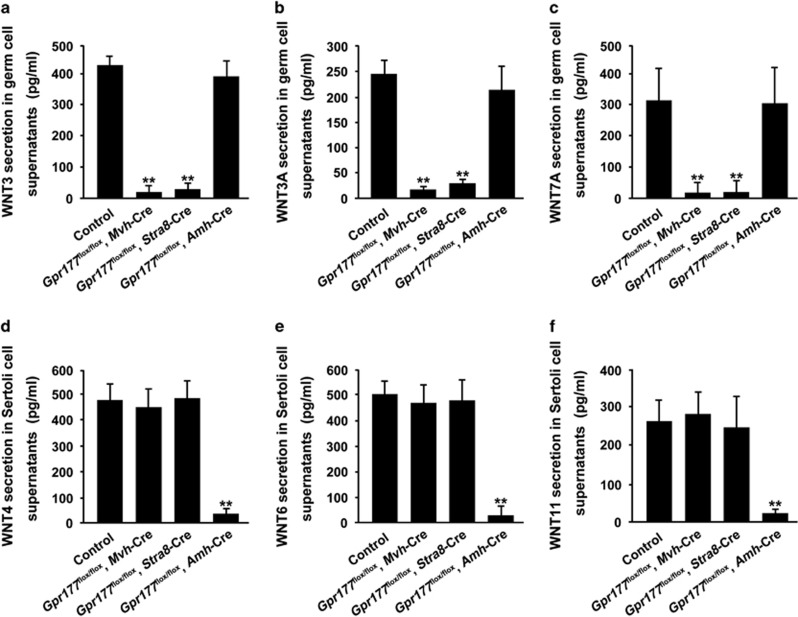
Loss of *Gpr177* blocked the extracellular secretion of WNT proteins. (**a–c**) Protein levels of WNT3 (**a**), WNT3A (**b**) and WNT7A (**c**) in germ cell culture supernatants from control, *Gpr177*^flox/flox^, *Mvh*-Cre; *Gpr177*^flox/flox^, *Stra8*-Cre and *Gpr177*^flox/flox^, *Amh*-Cre testes. (**d**–**f**) Secreted WNT4 (**d**), WNT6 (**e**) and WNT11 (**f**) examined by ELISA methods were observed in supernatants of Sertoli cells from control and three *Gpr177* cKO testes. The data are expressed as the mean±S.E.M. **P<0.01

WLS/GPR177 is required for the secretion of WNT proteins from signalling cells. We postulated that selective loss of *Gpr177* in germ cells and Sertoli cells would block the secretion of germ cell-specific and Sertoli cell-specific WNT proteins, respectively. Thus, extracellular secretion of WNT proteins, as examined by enzyme-linked immunosorbent assay (ELISA), was detected in supernatants of germ cells or Sertoli cells from control and *Gpr177* cKO testes. Using qRT-PCR, we observed that *Wnt3*, *Wnt3a* and *Wnt7a* were predominantly expressed in germ cells (Supplementary Figures S1a and b), while Sertoli cells mainly expressed several *Wnts*, including *Wnt4*, *Wnt6* and *Wnt11* (Supplementary Figures S1a and c), which is consistent with previous studies.^10, 29^ We observed that secreted protein levels of WNT3, WNT3A and WNT7A were significantly decreased in culture supernatants of germ cells from two germ cell-specific *Gpr177* knockout testes, but not from *Gpr177*^flox/flox^, *Amh*-Cre testes (Figures 3a–c). In addition, loss of *Gpr177* in Sertoli cells inhibits extracellular secretion of WNT4, WNT6 and WNT11 by Sertoli cells (Figures 3d–f).

### Normal spermatogenesis in *Gpr177* cKO mice at 8 weeks

**Figure 4:**
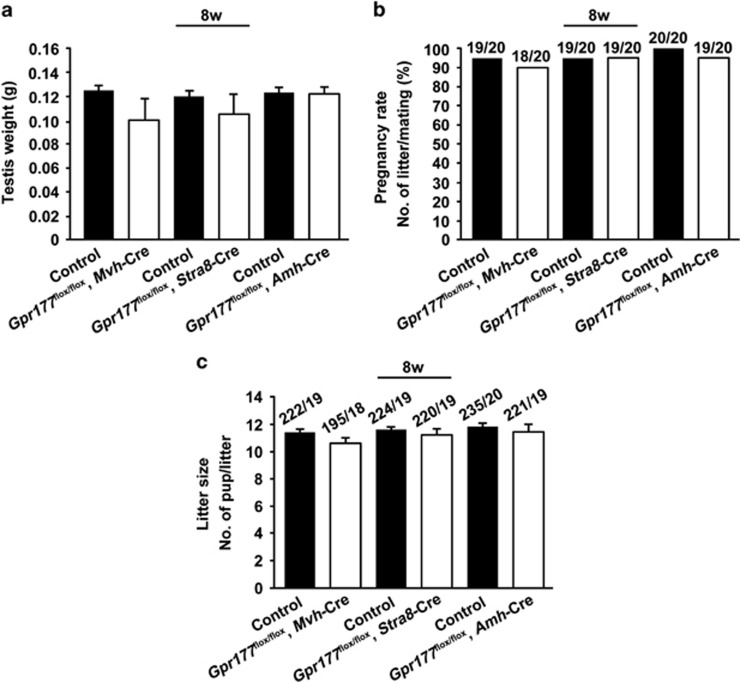
Eight-week-old *Gpr177* cKO male mice were fertile. (**a**) Testis weights were examined. The data are expressed as the mean±S.E.M. (**b**) The pregnancy rate was calculated as the ratio of the number of pregnant females to the number of successfully mating females. (**c**) When calculating the average litter size, only the females that generated pups were included. The data are expressed as the mean±S.E.M.

**Figure 5:**
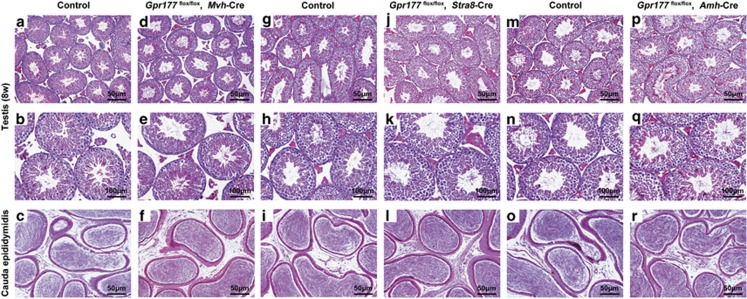
Testis and cauda epididymis morphology of 8-week-old Gpr177 cKO mice. (**a**–**r**) Testicular and cauda epididymal sections stained with H&E. No overt morphological abnormalities were observed in *Gpr177*^flox/flox^, *Mvh*-Cre (**d**–**f**), *Gpr177*^flox/flox^, *Stra8*-Cre (**j**–**l**) and *Gpr177*^flox/flox^, *Amh*-Cre (**p**–**r**) mice relative to their respective littermate controls (**a**–**c**, **g**–**i**, **m**–**o**). The images shown are representative results of experiments that were repeated three times using samples from different sets of testes, which yielded similar results. Scale bars in the first and third lines, 50 *μ*m. Scale bars in the second line, 100 *μ*m

After successfully generating two germ cell-specific *Gpr177* knockout mouse models, we investigated the role of GPR177 in germ cells. In 8-week-old cKO mice, there were no overt abnormalities in testis weight (127±2 mg in wild-type mice and 110±7 mg in *Gpr177*^flox/flox^, *Mvh*-Cre mice; 122±2 mg in wild-type mice and 114±6 mg in *Gpr177*^flox/flox^, *Stra8*-Cre mice) (Figure 4a), pregnancy rate (95% in wild-type mice and 90% in *Gpr177*^flox/flox^, *Mvh*-Cre mice; 95% in wild-type mice and 95% in *Gpr177*^flox/flox^, *Stra8*-Cre mice) (Figure 4b) and litter size (11.6±0.2 in wild-type mice and 10.7±0.3 in *Gpr177*^flox/flox^, *Mvh*-Cre mice; 11.8±0.2 in wild-type mice and 11.4±0.3 in *Gpr177*^flox/flox^, *Stra8*-Cre mice) (Figure 4c). Furthermore, *Gpr177*^flox/flox^, *Mvh*-Cre testes (Figures 5d–f), *Gpr177*^flox/flox^, *Stra8*-Cre testes (Figures 5j–l) and their respective littermate control testes (Figures 5a–c, g–i) exhibited typical seminiferous tubule morphology with all stages of spermatogenic cells (from spermatogonia to spermatozoa) at 8 weeks, indicating that spermatogenesis was normal in germ cell-specific *Gpr177* knockout males. The same conclusion was drawn from the immunofluorescence results of staining germ cell-specific marker MVH in the testes and spermatozoa-specific marker AQP3 in the cauda epididymis of *Gpr177*^flox/flox^, *Mvh*-Cre males (Supplementary Figures S2b and h) and *Gpr177*^flox/flox^, *Stra8*-Cre males (Supplementary Figures S2d and j) at 8 weeks.

To test whether Sertoli cell-specific *Gpr177* deletion causes defects in fertility, we bred *Gpr177*^flox/flox^, *Amh*-Cre males with wild-type females. The testis weight (126±2 mg in wild-type mice and 124±3 mg in *Gpr177*^flox/flox^, *Amh*-Cre mice), pregnancy rate (100% in wild-type mice and 95% in *Gpr177*^flox/flox^, *Amh*-Cre mice) and number of pups per litter (11.9±0.2 in wild-type mice and 11.7±0.3 in *Gpr177*^flox/flox^, *Amh*-Cre mice) were not significantly different between *Gpr177*^flox/flox^, *Amh*-Cre males and their respective littermate control males at 8 weeks (Figures 4a–c), indicating normal fertility of *Gpr177*^flox/flox^, *Amh*-Cre male mice. Furthermore, histological and immunofluorescence analysis of *Gpr177*^flox/flox^, *Amh*-Cre testes did not identify any major structural defects in testis morphology and spermatogenesis (Figures 5p–r and Supplementary Figures S2f and l).

### Late-onset testicular atrophy and fertility decline in germ cell-specific *Gpr177* deletion mice

**Figure 6:**
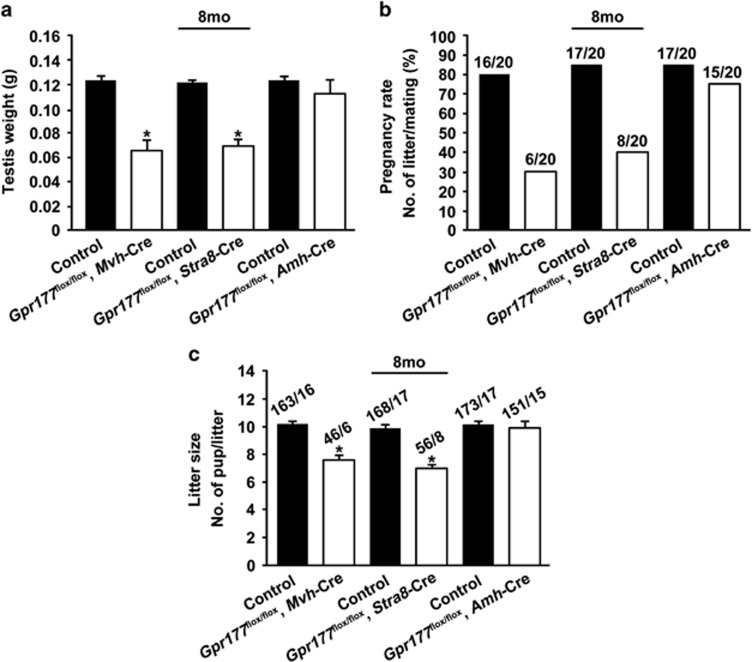
Eight-month-old germ cell-specific *Gpr177* knockout male mice were subfertile. (**a**) Testis weights were examined. (**b**) The pregnancy rate was calculated as the ratio of the number of pregnant females to the number of successfully mating females. (**c**) When calculating the average litter size, only the females that generated pups were included. The data are expressed as the mean±S.E.M. *P<0.05

**Figure 7:**
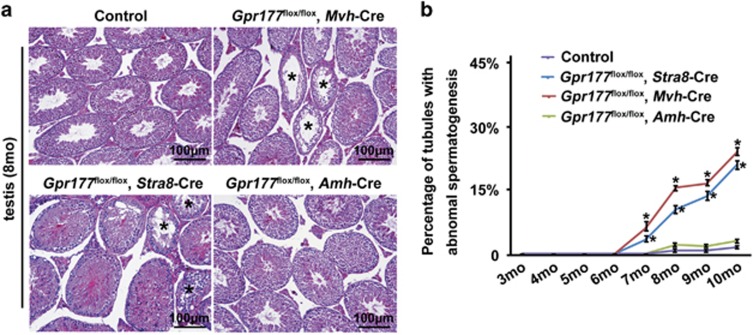
Testis morphology of 8-month-old *Gpr177* cKO mice. (**a**) Testicular sections were stained with H&E. Stars indicated tubules with abnormal spermatogenesis. The images shown were representative results of experiments that were repeated three times using samples from different sets of testes, which yielded similar results. Scale bars, 100 *μ*m. (**b**) The percentage of tubules with abnormal spermatogenesis was counted from month 3 to month 10. The data are expressed as the mean±S.E.M. *P<0.05

The spermatogenesis and fertility of *Gpr177*^flox/flox^, *Mvh*-Cre and *Gpr177*^flox/flox^, *Stra8*-Cre mice were indistinguishable from those of control mice at 8 weeks (above, Figures 4 and 5). However, these two germ cell-specific *Gpr177* knockout mice developed age-dependent testicular atrophy. By 8 months of age, the average weight of a *Gpr177*^flox/flox^, *Mvh*-Cre testis was approximately 59% of that of a control testis (124±2 mg in wild-type mice and 73±4 mg in *Gpr177*^flox/flox^, *Mvh*-Cre mice) (*P*<0.05), while the average weight of a *Gpr177*^flox/flox^, *Stra8*-Cre testis was approximately 62% of that of a control testis (122±1 mg in wild-type mice and 75±3 mg in *Gpr177*^flox/flox^, *Stra8*-Cre mice) (*P*<0.05) (Figure 6a). Testicular atrophy in the cKO mice was accompanied by a significant decline in pregnancy rate (80% in control mice and 30% in *Gpr177*^flox/flox^, *Mvh*-Cre mice; 85% in wild-type mice and 40% in *Gpr177*^flox/flox^, *Stra8*-Cre mice) (Figure 6b) and litter size (10.3±0.2% in wild-type mice and 7.6±0.3% in *Gpr177*^flox/flox^, *Mvh*-Cre mice; 9.8±0.3% in wild-type mice and 7.0±0.3% in *Gpr177*^flox/flox^, *Stra8*-Cre mice) (Figure 6c) at 8 months. Observation of the mating behaviour of *Gpr177* cKO males showed that they copulated with females at a rate comparable to control males, which indicated that behavioural factors were not the cause of the reduced fertility. H&E staining examination of the seminiferous epithelium of *Gpr177* cKO mice revealed some histological abnormalities. The most obvious of these abnormalities was epithelial vacuolisation in some tubules, which were devoid of spermatocytes and spermatids and were evident in 8-month-old *Gpr177* cKO mice (Figure 7a). As the mouse age increased, the percentage of tubules with abnormal spermatogenesis significantly increased in *Gpr177*^flox/flox^, *Mvh8*-Cre males (6.2±0.8% at 7 months, 15.5±0.4% at 8 months, 16.9±0.4% at 9 months and 24.2±0.6% at 10 months) and *Gpr177*^flox/flox^, *Stra8*-Cre males (3.2±0.4% at 7 months, 11.1±0.6% at 8 months, 13.8±0.8% at 9 months and 20.3±0.8 at 10 months), compared with their controls (0% at 7 months, 2.5±0.2% at 8 months, 2.1±0.2% at 9 months and 3.6±0.2% at 10 months) (Figure 7b). In contrast, we did not observe obviously abnormal spermatogenesis or fertility decline even in aged (7- to 10-month-old) *Gpr177*^flox/flox^, *Amh*-Cre mice (Figures 6 and 7).

### Oxidative stress is involved in age-dependent spermatogenic damage

**Figure 8:**
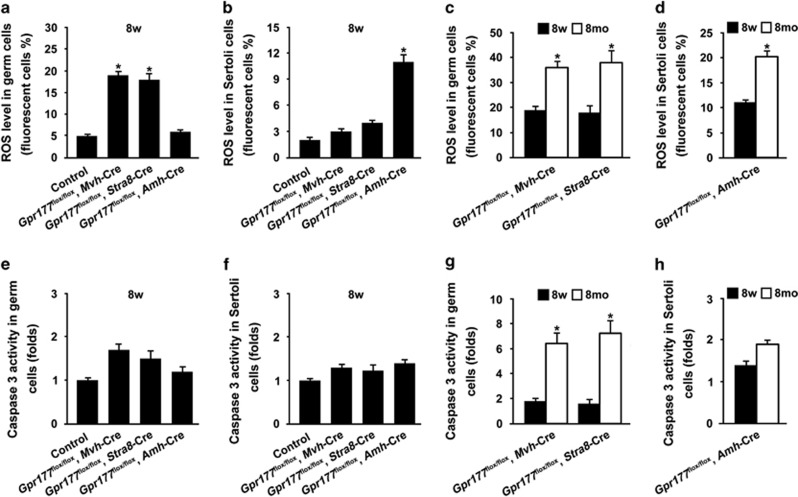
Status of ROS and apoptosis in germ and Sertoli cells from 8-week-old and 8-month-old *Gpr177* testes. (**a**) ROS level in germ cells from control, *Gpr177* cKO testes at 8 weeks. (**b**) ROS level in Sertoli cells from control, *Gpr177* cKO testes at 8 weeks. (**c**) ROS level in germ cells from 8-week-old and 8-month-old germ cell-specific *Gpr177* knockout testes. (**d**) ROS level in Sertoli cells from 8-week-old and 8-month-old *Gpr177*^flox/flox^, *Amh*-Cre knockout testes. (**e**) Flow cytometric analysis of Caspase 3 activity in germ cells from control, *Gpr177* cKO testes at 8 weeks. (**f**) Caspase 3 activity in Sertoli cells from control, *Gpr177* cKO testes at 8 weeks. (**g**) Caspase 3 activity in germ cells from 8-week-old and 8-month-old germ cell-specific *Gpr177* knockout testes. (**h**) Caspase 3 activity in Sertoli cells from 8-week-old and 8-month-old *Gpr177*^flox/flox^, *Amh*-Cre knockout testes. The data are expressed as the mean±S.E.M. *P<0.05

Given that elevation of reactive oxygen species (ROS) impairs spermatogenesis in an age-dependent manner, we examined the status of ROS and apoptosis in different cell types from control and *Gpr177* cKO testes at both 8 weeks and 8 months. As shown in Figure 8a, a significant increase of ROS level was observed in germ cells from *Gpr177*^flox/flox^, *Mvh*-Cre and *Gpr177*^flox/flox^, *Stra8*-Cre testes at 8 weeks, compared with germ cells from control and *Gpr177*^flox/flox^, *Amh*-Cre testes at the same age. Selective loss of *Gpr177* in Sertoli cells promoted a significant increase of ROS level in Sertoli cells at 8 weeks (Figure 8b). These data suggest that blocking WNT secretion by *Gpr177* cKO causes increase of oxidative stress in corresponding cells from adult (8-week-old) testes. Furthermore, elevation of ROS exhibited an age-dependent manner. We observed that the ROS level was significantly increased in germ cells (Figure 8c) and Sertoli cells (Figure 8d) from 8-month-old *Gpr177* cKO testes, compared with cells from 8-week-old *Gpr177* cKO testes. The activity of Caspase 3 in germ cells (Figure 8e) and Sertoli cells (Figure 8f) was similar between control and *Gpr177* cKO testes at 8 weeks. However, selective loss of *Gpr177* in germ cells caused significant increase in the activity of Caspase 3 at 8 months relative to 8 weeks (Figure 8g). In contrast, the activity of Caspase 3 in Sertoli cells from 8-month-old *Gpr177*^flox/flox^, *Amh*-Cre testes was not statistically different from *Gpr177*-deficient Sertoli cells at 8 weeks (Figure 8h).

## Discussion

Cell–cell communication via WNT signalling represents a fundamental means by which animal development and homoeostasis are controlled. Components of the cellular machinery responsible for transducing WNT signals from the cell surface to the nucleus, which is mainly mediated by *β*-catenin, have been identified in receiving cells, but the identification of components associated with the events occurring in WNT-secreting cells is incomplete (reviewed by Logan and Nusse^1^). Importantly, recent studies have identified a novel WNT pathway component, Wntless (WLS), that promotes WNTs secretion from WNT-producing cells into the extracellular milieu.^32, 33^ WLS is evolutionarily and functionally conserved; seven-pass membrane protein, intriguingly, acts exclusively in WNT signal-sending cells. Accordingly, *Gpr177* (mouse orthologue of *Drosophila Wls*) cKO mice are excellent models to study the role of WNT singalling (both canonical and noncanonical) and total WNTs.

Mice mutant for *β*-catenin using other Cre drivers have phenotypes that are inconsistent with each other. Germ cell-specific *β*-catenin knockout mediated by *Stra8*-Cre, which is expressed in differentiating spermatogonia at the onset of differentiation, did not cause a detectable phenotype.^28^ However, spermatid-specific *β*-catenin knockout mediated by *Prm1*-Cre has been shown to cause impaired fertility as a result of reduced sperm counts.^26^ Kerr *et al.*^44^ reported disrupted spermatogenesis in both loss- and gain-of-WNT signalling function experiments using *Ah*Cre. In a recent article, Takase *et al.*^29^ reported that WNT6 secreted by Sertoli cells activates WNT/*β*-catenin signalling and mediates the proliferation of undifferentiated spermatogonia. These researchers demonstrated that undifferentiated spermatogonia are WNT-responsive cells by taking advantage of genetic lineage tracing using the WNT target gene *Axin2*. We hypothesise that undifferentiated spermatogonia are not the only WNT-responsive cell population within the testes, because previous studies have demonstrated the expression and conditional deletion *β*-catenin in meiotic and post-meiotic germ cells.^26, 28^ Furthermore, the *Axin2*-LacZ reporter line used in the current study revealed different WNT-responsive cells than the *Tcf/Lef*-LacZ mouse reporter line.^7^ Conditional deletion of *β*-catenin in AXIN2-expressing cells upon tamoxifen injection reduced the proliferation of undifferentiated spermatogonia. Thus, the authors suggested that WNT/*β*-catenin signalling promotes stem cell proliferation. However, we hypothesise that the reduced proliferation could also be due to impaired cadherin-mediated adherens junctions rather than disrupted WNT/*β*-catenin signalling, based on the following evidence. First, although *β*-catenin plays a central role in the canonical WNT pathway, it also serves as a membrane protein in the cell junction complex.^45, 46^ Second, we found in current study that conditional deletion of the *Gpr177* gene in Sertoli cells using *Amh*-Cre (blocking WNTs secretion from Sertoli cells) had no effect on spermatogenesis and male fertility. Additionally, the evidence that Sertoli cells secrete WNT6 as a paracrine signal for undifferentiated spermatogonia is insufficient. In addition to Sertoli cells, other somatic cell types contribute to the SSC niche, including Leydig cells,^47^ peritubular myoid cells^48^ and peritubular macrophages.^49^ Thus, WNT-producing cells are not limited to Sertoli cells. *Wnt6* was shown to be specifically expressed in Sertoli cells, particularly in the basal compartment. However, using qRT-PCR, we observed that Sertoli cells express several *Wnts*, including, but not limited to, *Wnt6* (Supplementary Figure S1). The generation of Sertoli cell-specific *Wnt6* knockouts would be helpful to confirm this conclusion. Thus, these data are insufficient to draw the conclusion that Sertoli cells secrete WNT6 to activate canonical WNT signalling in undifferentiated spermatogonia.

Our study demonstrates that GPR177 in Sertoli cells has no apparent influence on spermatogenesis, whereas germ cell-specific *Gpr177* deletion mice exhibit an age-dependent reproductive phenotype: fertile when young and subfertile when older. We suggest that accumulated WNTs do harm to germ cells and oxidative stress and apoptosis are involved in age-dependent spermatogenic damage of germ cell-specific *Gpr177* deletion mice. ROS has a dual role in reproductive systems, both beneficial and harmful depending on their nature and concentration as well as location and length of exposure.^50, 51^ The decline in male fertility with aging is associated with increasing oxidative damage in the male reproductive system. Mice lacking nuclear factor-erythroid 2-related factor 2 (*Nrf2*), or superoxide dismutase 1 (*Sod1*), or inner mitochondrial membrane peptidase 2-like (*Immp2l*) develop impaired spermatogenesis in an age-dependent manner.^52, 53, 54^ In contrast, SSCs depleted of ROS stop proliferating, while enhanced self-renewal is observed when ROS levels are increased.^55^ Thus, ROS levels need to be tightly controlled in germ cells. In this study, we suggest that elevation of ROS level in germ cells triggers apoptotic signalling to disrupt spermatogenesis in aged (8-month-old) *Gpr177*-deficient germ cells. The reason why young (8-week-old) germ cell-specific *Gpr177* knockout mice (germ cells also endure oxidative stress) exhibit normal spermatogenesis is still unclear. One of the main reasons is that ROS level in 8-week-old *Gpr177*-deficient germ cells was significantly lower than that in 8-month-old mutant germ cells (Figure 8c). Compared with germ cells, Sertoli cells seem to endure oxidative stress to a certain extent, because we did not observe significant apoptosis in even 8-month-old *Gpr177*-deficient Sertoli cells (Figures 8d and h).

Notably, two recent studies suggest that mammalian spermatozoa respond to WNT signals released from the epididymis and WNT signalling controls sperm maturation independent of *β*-catenin.^56, 57^ It is a novel way of WNT signalling in regulating spermatogenesis. We suggest that generation of cKO mice in which *Gpr177* was specifically knocked out in epididymal cells (using *Rnase10*-Cre or *Lcn5*-Cre) could provide further evidence whether GPR177-mediated WNT secretion from epididymal cells act as an epididymal sperm maturation signal.

## Material and Methods

### Mice

All animal works were carried out in accordance with the protocols approved by the Institutional Animal Care and Use Committee at the Institute of Zoology (IOZ), Chinese Academy of Sciences (CAS). All the mice were maintained in a C57BL/6;129/SvEv mixed background. *Gpr177*^flox/flox^ mice homozygous for a floxp allele of *Gpr177* (The Jackson Laboratory, Bar Harbor, ME, USA, stock no. 012888), *Mvh*-Cre (The Jackson Laboratory, stock no. 006954), *Stra8*-Cre (The Jackson Laboratory, stock no. 008208) and *Amh*-Cre (The Jackson Laboratory, stock no. 007915) mice were used in the present study and were described previously.^27, 38, 58, 59^ *W/W*^*v*^ mice were introduced from The Jackson Laboratory (stock no. 000693) and SSC transplantation was performed as described previously.^60^

### Fertility rate

Two- and eight-month-old *Gpr177*^flox/flox^, *Mvh*-Cre, *Gpr177*^flox/flox^, *Stra8*-Cre and *Gpr177*^flox/flox^, *Amh*-Cre males and their respective littermate controls were separately housed with wild-type C57BL/6 females (ratio=1:2) for 3 months. The pregnancy rate (no. of litter/mating) and litter size (no. of pup/litter) was recorded in each group. All mice were housed under controlled photoperiod conditions and supplied with food and ddH_2_O *ad libitum*.

#### Isolation of Sertoli, interstitial and spermatogenic cells

The method to isolate cells from the testes of adult mice was modified slightly based on previous reports.^61, 62^ Briefly, tubules without tunica albuginea were incubated in 1 mg/ml BSA containing 1mg/ml collagenase IV (Sigma-Aldrich, St. Louis, MO, USA) and under shaking (100 r.p.m.) for 20 min in a 37 °C water bath. Tubules were collected by centrifugation at 40 × *g*, and the crude cell suspension was filtered through a 200-mesh nylon membrane. The cell suspension was separated in a discontinuous Percoll (Pharmacia, Shanghai, China) gradient of 30, 40, 50 and 60% at 800 × *g* for 20 min. A gradient fraction mainly containing interstitial cells between 50 and 60% layers (1.067–1.077 g/ml) was collected, washed once in culture medium and then plated in DMEM/F12. For germ cell and Sertoli cell isolation, precipitated seminiferous tubules mentioned above were dissociated and incubated with 1mg/ml collagenase IV, 1 mg/ml hyaluronidase, 1 mg/ml trypsin and 0.5 mg/ml DNase I (Sigma-Aldrich) in DMEM/F12 medium for 15 min at 37 °C in a shaker. Dispersed cells were centrifuged at 500 × *g* for 5 min at 4 °C and washed twice. Then, cell suspension was cultured in DMEM/F12 medium supplemented with penicillin (100 UI/ml), streptomycin (100 *μ*g/ml) and 10% FBS. This technique called panning is extremely efficient for the gross separation and purification of the germ cells. Sertoli cells were treated with a hypotonic solution (20 mM Tris, pH 7.4) for 1 min to remove the residual germ cells. Collect germ cells from suspension in culture medium and gently overlay the top of the prepared BSA gradient^63^ with the cell suspension. The gradient was centrifuged at 800 × *g* for 30 min at 4 °C. The fractions at the 25–32% interface were collected. Cells were cultured at 34 °C in a mixture of 5% CO_2_:95% air for 18 h.

### Histological examination and immunofluorescence

The control and *Gpr177* cKO male mice were killed via cervical dislocation and the testes were immediately fixed in Bouin's solution for hematoxylin and eosin (H&E) staining or in 4% formaldehyde (PFA) in PBS for immunofluorescence, as previously described.^61, 64^ In brief, tissue sections were deparaffinised and rehydrated, followed by antigen retrieval in 10 mM sodium citrate buffer. The sections were blocked using a blocking buffer (donkey serum, 0.3% Triton X-100 in PBS) and incubated with primary antibodies against GPR177 (1:200; Santa Cruz, St. Louis, MO, USA, sc-133635), MVH (1:300; Abcam, Cambridge, UK, ab13840) or AQP3 (1:400; kind gift from Dr. Qi Chen) overnight at 4 °C. Sections were washed and incubated with FITC-conjugated secondary antibodies (1:200; Jackson ImmunoResearch, West Grove, PA, USA) for 1 h and counterstained with DAPI (1:1000; Sigma-Aldrich) to identify the nuclei.

### Quantitative (q)RT-PCR

RNA was extracted using Trizol (Invitrogen, Dallas, TX, USA) according to the manufacturer's protocol. RNA samples were subjected to reverse transcription using a PrimeScript RT Reagent Kit (Takara, Dalian, China). The reactions were run in triplicate in three independent experiments. The CT values for the samples were normalised to the corresponding *Gapdh* CT values, and relative expression levels were calculated using the ^ΔΔ^CT method. The primer pair for *Gpr177*: forward (5′-TGGGAAGCAGTCTAGCCTCC-3′) and reverse (5′-GCAGCACAAGCCAAGGTGATA-3′). The primer pair for *Gapdh*: forward (5′-AGGTCGGTGTGAACGGAT-3′) and reverse (5′-TGTAGACCATGTAGTTGA-3′).

### Western blot

Western blot analysis was performed as described previously.^61^ Briefly, proteins were electrophoresed under reducing conditions in 10% SDS-PAGE gels and transferred to nitrocellulose membranes. The blots were blocked in 5% BSA and incubated overnight at 4 °C with the primary antibody, followed by incubation with the secondary antibody (anti-rabbit Dye 800CW; LI-COR, St. Louis, MO, USA) for 1 h at room temperature. The specific signals and the corresponding band intensities were evaluated using an Odyssey Infrared Imaging system (Odyssey, Berlin, Germany). The protein level was normalised and plotted against *β*-tubulin. The following antibodies were used in this study: rabbit anti-GPR177 (1/800; Santa Cruz, sc-133635) and rabbit anti-*β*-tubulin (1/3000; Abcam, 6046).

### ELISA

The germ cells and Sertoli cells were isolated from control and *Gpr177* cKO testes and cultured in DMEM/F12 with 10% serum as described above. Supernatants were collected and used for ELISA analysis for quantification of secreted WNT3 (CSB-EL026135MO), WNT3A (CSB-EL026136MO), WNT7A (CSB-EL026141MO), WNT4 (CSB-EL026137MO), WNT6 (CSB-EL026140MO) and WNT11 (CSB-EL026131MO) (CUSABIO, Wuhan, China) using the manufacturer's instructions.

### ROS assays

The generation of ROS in germ cells and Sertoli cells from control and *Gpr177* cKO testes was measured using 5, and 6-chloromethyl-2′,7-dichlorodihydrofluorescein diacetate ethyl ester (DCFH-DA) (Invitrogen, Carlsbad, CA, USA) at a concentration of 5 mM for 30 min. This ester diffuses into cells where it is cleaved and trapped inside the cells as DCFH. DCFH is oxidised by ROS to 2′,7′-dichlorofluorescein, which can be easily detected by its strong fluorescence.^65^ After washing the cells three times with PBS, the conversion of DCFH to dichlorofluorescein (DCF; green fluorescence) was measured using a flow cytometer (BD FACS Calibur; BD Pharmingen, St. Louis, MO, USA). Data were expressed as the percentage of the fluorescent cells.

### Analysis of Caspase 3 activity

Caspase 3 activity was determined using PE active caspase 3 apoptosis kit (BD Pharmingen). Germ cells or Sertoli cells from control and *Gpr177* cKO testes were resuspended in 0.5 ml Cytofix/Cytoperm solution for 20 min on ice and then incubated in 100 *μ*l of Perm/Wash buffer containing 20 *μ*l Caspase 3 antibody for 30 min at room temperature. Each sample was then added with 400 *μ*l Perm/Wash buffer, and Caspase 3 activity signals were analysed by flow cytometry (BD FACS Calibur; BD Pharmingen).

### Statistical analysis

Protein and mRNA levels, testis weights, ROS levels, Caspase 3 activity, fertility rate, litter size and percentage of tubules with abnormal spermatogenesis between control and *Gpr177* cKO mice were analysed by using the Student's *t*-test. Results are presented as mean±S.E.M. Statistical significance was set at *P<0.05; **P<0.01.

## Abbreviations

WNT: wingless-related MMTV integration site
ROS: reactive oxygen species
PGC: primordial germ cell
SSC: spermatogonial stem cell
cKO: conditional knockout
E: embyonic day
PND: postnatal day
H&E: hematoxylin and eosin
PFA: formaldehyde
DCFH-DA: 5, and 6-chloromethyl-2′,7-dichlorodihydrofluorescein diacetate ethyl ester.
