# Supplementary figures and images for: Does murine spermatogenesis require WNT signalling? A lesson from Gpr177 conditional knockout mouse models
Source: Cell Death Dis. 2016 Jun 30;7(6):e2281–. doi: 10.1038/cddis.2016.191 (PMC5108341; doi:10.1038/cddis.2016.191)

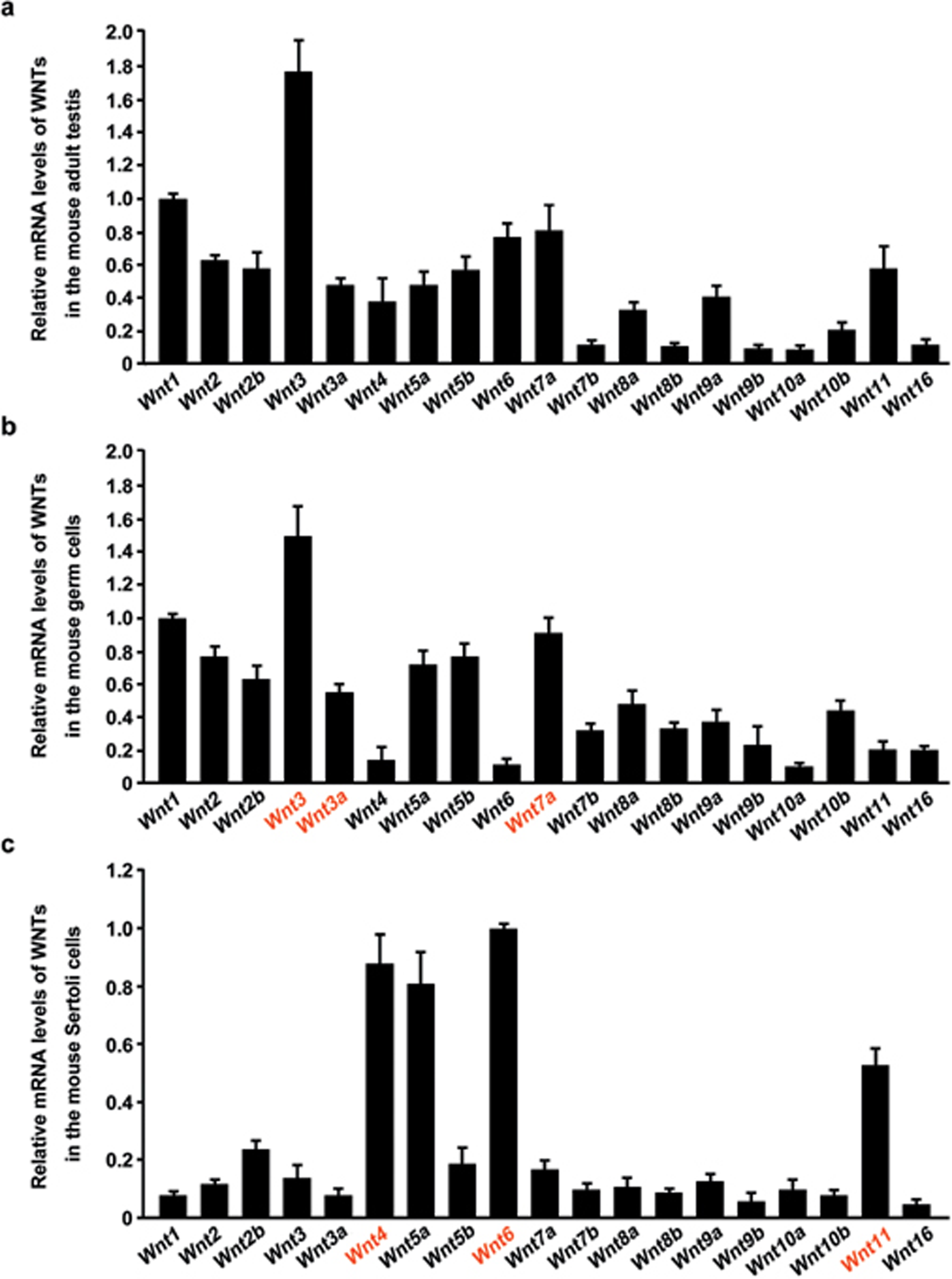

Supplement: Supplementary Figure 1 [file cddis2016191x1.tif]

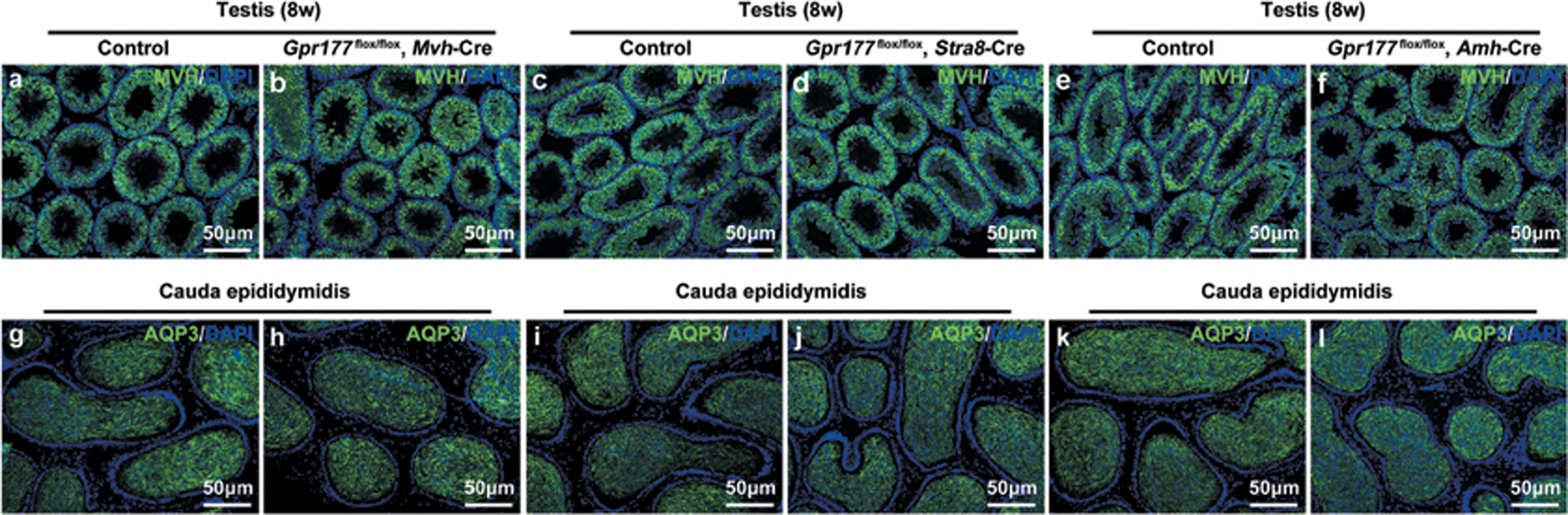

Supplement: Supplementary Figure 2 [file cddis2016191x2.tif]
