# Supplementary material for: Does murine spermatogenesis require WNT signalling? A lesson from Gpr177 conditional knockout mouse models
Source: Cell Death Dis. 2016 Jun 30;7(6):e2281–. doi: 10.1038/cddis.2016.191 (PMC5108341; doi:10.1038/cddis.2016.191)
Supplement: Supplementary Figure Legends [file cddis2016191x3.docx]

**Figure S1** Expression level of WNT ligands in the adult mouse testes (**a**), isolated germ cells (**b**) and Sertoli cells (**c**) evaluated by qRT-PCR. *Gapdh* served as the internal control gene. Data are presented as mean ± SEM.

**Figure S2** Spermatogenesis in 8-week-old *Gpr177* cKO mice. (**a**-**f**) Immunofluorescence staining of MVH (a germ cell marker) in 8-week-old *Gpr177* cKO testis. (**g**-**i**) Immunofluorescence staining of AQP3 (a spermatozoa marker) in 8-week-old *Gpr177* cKO cauda epididymis. The images shown were representative results of experiments that were repeated three times, which yielded similar results. Scale bars, 50 *μ*m.
